# Anti-Inflammatory Therapies for Treatment of Inflammation-Related Preterm Brain Injury

**DOI:** 10.3390/ijms22084008

**Published:** 2021-04-13

**Authors:** Jaya D. Prasad, Katherine C. Gunn, Joanne O. Davidson, Robert Galinsky, Scott E. Graham, Mary J. Berry, Laura Bennet, Alistair J. Gunn, Justin M. Dean

**Affiliations:** 1 Department of Physiology, Faculty of Medical and Health Sciences, University of Auckland, 85 Park Road, Grafton, Auckland 1010, New Zealand; j.prasad@auckland.ac.nz (J.D.P.); K.gunn@nucleusnetwork.com.au (K.C.G.); joanne.davidson@auckland.ac.nz (J.O.D.); l.bennet@auckland.ac.nz (L.B.); aj.gunn@auckland.ac.nz (A.J.G.); 2 The Ritchie Centre, Hudson Institute of Medical Research, Clayton, VIC 3168, Australia; robert.Galinsky@hudson.org.au; 3 Department of Molecular Medicine and Pathology, Faculty of Medical and Health Sciences, University of Auckland, Auckland 1010, New Zealand; s.graham@auckland.ac.nz; 4 Department of Pediatrics and Health Care, University of Otago, Dunedin 9016, New Zealand; max.berry@otago.ac.nz

**Keywords:** white matter injury, infection, inflammation, neuroprotection

## Abstract

Despite the prevalence of preterm brain injury, there are no established neuroprotective strategies to prevent or alleviate mild-to-moderate inflammation-related brain injury. Perinatal infection and inflammation have been shown to trigger acute neuroinflammation, including proinflammatory cytokine release and gliosis, which are associated with acute and chronic disturbances in brain cell survival and maturation. These findings suggest the hypothesis that the inhibition of peripheral immune responses following infection or nonspecific inflammation may be a therapeutic strategy to reduce the associated brain injury and neurobehavioral deficits. This review provides an overview of the neonatal immunity, neuroinflammation, and mechanisms of inflammation-related brain injury in preterm infants and explores the safety and efficacy of anti-inflammatory agents as potentially neurotherapeutics.

## 1. Introduction

Preterm birth is a major cause of neonatal mortality and morbidity. Worldwide, the rates of preterm birth range between 6–15% [1,2]. Although the survival of preterm infants has markedly increased because of improved neonatal intensive care, these babies have high rates of brain injury and disability, with the greatest risk at earliest gestational ages [3,4]. Cerebral palsy occurs in 5–10% of preterm infants born <32 weeks gestation [5,6]. However, as many as 50% of extremely preterm infants (<28 weeks gestation) also exhibit subtle but persisting adverse neurodevelopmental outcomes, including neurobehavioral disturbances and intellectual disabilities related to learning, cognition, visuospatial integration, attention deficits, and socialization during childhood and later life [7,8]. As a result, the economic impact of preterm birth has been estimated as several times greater than that of all chronic neurodegenerative diseases, including Parkinson’s disease, largely because of life-long disability [9]. Thus, even a modest reduction in perinatal disability would have very large direct economic benefits and reduce the personal burden on affected individuals, their families, and society.

Although the causes are likely multifactorial, disability in preterm born children is strongly associated with infection and inflammation around the time of birth. Infection is implicated in 25–40% of all preterm births [10,11], while both intrauterine and postnatal infections are highly associated with brain injury and neurological impairment [12,13,14]. Given the apparently causal relationship between inflammation and adverse developmental outcomes in preterm born infants, it is logical to assume that modulation of the inflammatory response would be beneficial. However, this has not been clinically proven, and the current options for pharmacological intervention in neonates with inflammation-related brain injury remain very limited. 

Concerns arise, in part, because of the dual roles of inflammation in both normal physiology and pathophysiology. For example, the primary function of the immune response is to help limit the spread of injury and promote homeostasis at the site of injury, and many cytokines are involved in normal brain development [15,16]. Nevertheless, sustained, uncontrolled immune activation can lead to further damage, as well as have severe consequences for the individual’s ability to cope with environmental exposures in later life [17,18]. Thus, designing neuroprotective therapies requires detailed knowledge of the evolving clinical phenotype and the cellular mechanisms in order to provide an effective balance between maintaining the normal physiological role of inflammation and reducing the pathological inflammatory response. Moreover, it is important to appreciate that the impact of exposure to infection or inflammation is highly dependent on their precise timing and duration. For example, in preterm fetal sheep, exposure to low levels of inflammation induced by an infusion of Gram-negative lipopolysaccharide (LPS) produced “self-tolerance”, leading to reduced death or injury during subsequent exposure to large boluses of LPS [19,20]. LPS exposure can also modulate injury from subsequent hypoxia-ischemia. In neonatal rodents, exposure to LPS can both dramatically sensitize and protect against hypoxic-ischemic brain injury, depending on the precise timing of LPS exposure; typically, either early or chronic exposure can sensitize to subsequent hypoxia-ischemia (e.g., [21,22]). In preterm fetal sheep, prolonged acute on chronic exposure to LPS was also reported to induce preconditioning and reduce the subsequent hypoxic-ischemic brain injury [23,24]. This can also affect other organs. For example, in preterm fetal sheep, chorioamnionitis induced by intra-amniotic injection of LPS induced evidence of endotoxin tolerance in the fetal lung [25]. 

This review summarizes the current understanding of the role of the immune system during neonatal infection and the key inflammatory pathways proposed to underlie brain injury after early life exposure to infection/inflammation and then critically examines current evidence for use of anti-inflammatory therapies for the treatment of inflammation-related preterm brain injury.

## 2. The Neonatal Immune System

Development of the immune system involves a number of changes that occur during the first few years of life. Compared with adults, the neonatal immune system is considered to be immature in its functionality, which increases the susceptibility to infection, a phenomenon that is enhanced by prematurity [26]. Throughout pregnancy, the immune system of the fetus is dependent on the fetal–maternal interface, where fetal survival requires significant regulation of the maternal immune system without compromising the maternal immune protection. As a result, the neonatal infant has an immune system that has not fully matured around the time of birth, exhibiting diminished innate and adaptive responses and a relatively limited immunological memory [27,28]. This immunological profile provides the fetus with an intrauterine environment that enables immune tolerance to maternal antigens and a lack of antigen-priming exposures, which skews them towards immune tolerance (T-helper (Th)2) rather than towards defense from invading pathogens (Th1) [29,30].

### Protection from Pathogens

Protection against pathogens is achieved by coordinated actions of the innate and the adaptive arms of the immune system, where innate immune responses are the first line of host defense. In newborn infants, the innate immune system is largely responsible for mediating immune responses, as the adaptive immune responses (responsible for eliminating specific pathogens) are relatively immature [26,29]. However, at birth, the innate immune system is also considered to be immature, exhibiting impaired or depressed functionality. This is characterized by a decreased ability to produce inflammatory cytokines (e.g., tumor necrosis factor alpha (TNF-α) or interleukin-1 beta (IL-1β)) [31,32] and a smaller pool of immune cells (monocytes and neutrophils) that have an impaired ability to kill pathogens [33,34]. Importantly, this phenomenon is enhanced by prematurity. For example, compared with term born infants, preterm born infants have neutrophils with a decreased capacity to respond to bacterial stimulation [35], an impaired ability to migrate to sites of infection (i.e., diminished neutrophil rolling and expression of adhesion molecules) [36], and an impaired ability to clear debris from the site of infection, which can cause further aggravation at the sites of injury [37]. Preterm born infants also have monocytes with reduced pattern recognition receptors (PRRs) [38] and reduced cytokine production [39], resulting in dampened innate immune responses compared with infants at term.

With respect to the adaptive immune response, preterm born infants have reduced humoral protection, which is mainly provided from the mothers serum by transplacental passage during the third trimester of pregnancy [40]. In addition, preterm-born infants exhibit lower absolute numbers of circulating lymphocytes (B and T cells) compared with term-born infants [41]. How this affects the neonatal immune system remains unclear, as lymphocyte activation has been reported in preterm infants in response to immune activation [42]. Overall, the immaturity of the neonatal immune system, which is further enhanced with prematurity, reduces the capacity of the preterm infant to respond effectively to infection, which may increase their sensitivity to further injury.

## 3. From Systemic Inflammation to Neuroinflammation

Despite active communication between the central and peripheral immune responses, once peripheral inflammation is established, the immune privilege of the CNS can become severely undermined, resulting in widespread CNS immune responses. Peripheral immune stimulation results in a rapid inflammatory response, which is mediated by the PRRs (e.g., Toll-like receptors (TLRs)) of the innate immune system. These PRRs recognize pathogen-associated molecular patterns (PAMPs, i.e., viral or bacterial stimuli) and danger-associated molecular patterns (DAMPs, i.e., endogenous molecules released during cellular injury) [43]. The activation of these receptors triggers a cascade of intracellular signaling pathways (e.g., nuclear factor-κβ (NF-κβ) signaling and mitogen-activated protein kinase signaling), resulting in the production of pro- and anti-inflammatory cytokines and chemokines. Inflammatory cytokines also stimulate the production of other immune molecules (e.g., prostaglandins, C-reactive protein, and complement factors), which, in turn, stimulate the recruitment of neutrophils, macrophages, and leukocytes (e.g., lymphocytes) [44]. Importantly, as previously described, in newborn and premature infants, these immune cells exhibit reduced functionality, impairing their ability to remove debris from the site of infection [45]. 

Systemic inflammation can result in propagation of the immune response to the brain via a number of mechanisms. First, systemic cytokines can stimulate the production of metalloproteases, leading to disruption of the blood–brain barrier (BBB) and increased permeability to infiltrating immune cells [46,47]. Second, systemic cytokines and inflammatory molecules can enter the brain via the circumventricular organs or the choroid plexus, regions of the brain devoid of a BBB [48,49]. Third, cytokines and inflammatory molecules can enter the brain via saturable transporters that are responsible for the blood-to-brain influx of specific cytokines [50,51]. Together, once these infiltrating immune cells and cytokines enter the brain, they can propagate a central inflammatory response involving the local production of cytokines and gliosis (see Section 6). 

## 4. Causes of Perinatal Neuroinflammation

The causes of preterm brain injury and associated adverse neurological outcomes are complex and likely multifactorial [52]. Nevertheless, clinical and experimental studies suggest a key causative role for neuroinflammation. Inflammation can occur during the perinatal period or the postnatal period and can result from either infectious or noninfectious insults, as described below.

### 4.1. Intrauterine Infection

Intrauterine infection accounts for approximately 15% of term births [53], 30% of very preterm births (28–32 weeks of gestation) [54], and up to 60% of extremely preterm births (<28 weeks gestation) [55]. Chorioamnionitis is the most common form of intrauterine infection and is associated with white matter injury, an increased risk of cerebral palsy, poor neurodevelopmental outcomes, and overall disability [56,57]. Chorioamnionitis is characterized by inflammation of the chorion, amnion, and placenta, where bacterial invasion causes an acute inflammatory response of the placenta and/or fetal membranes [58]. Neonatal infants born to mothers with chorioamnionitis often develop fetal inflammatory response syndrome. This results from the fetus being in direct contact with infected amniotic fluid and/or inflammatory cells within the intrauterine environment and is associated with the elevated production of proinflammatory cytokines, which can induce injury to the developing brain [59,60] via the mechanisms described in Section 6.

### 4.2. Postnatal Infection

The injurious effects of infection and inflammation are not only limited to during pregnancy, with the risks for developing infection and subsequent brain injury being much higher in the postnatal period. For example, because of the increased number of invasive procedures associated with preterm birth and the pronounced immaturity of the immune system, preterm infants are at a significantly higher risk of developing at least one postnatal infection whilst in intensive care compared with their healthy term counterparts [13,54,61]. Importantly, the rate of infection increases with decreasing gestational age. For example, up to 25% of very preterm born infants [61] and up to 65% of extremely preterm born infants [13] were reported to contract a postnatal infection in the neonatal intensive care unit. Importantly, up to 50% of these infants exhibited neurodevelopmental impairments at two years of age, even when the infection was only evident clinically (i.e., without positive cultures) [62,63,64]. Repeated infections, recurrent inflammation (e.g., repeated inflammatory stimuli from mechanical ventilation), or a combination of antenatal infection followed by postnatal infection can further increase the risk of adverse neurodevelopmental outcomes [14,65,66].

### 4.3. Nonspecific/Sterile Inflammation

Inflammation-related preterm brain injury can also be induced in the absence of an infectious agent, where the peripheral and central inflammatory responses typically occur secondary to tissue damage (e.g., hypoxia-ischemia, intrauterine growth restriction, and mechanical ventilation). For example, hypoxia-ischemia can trigger both systemic and central inflammatory processes that continue for several weeks after the initial insult [18,67]. In human post-mortem studies of infants with hypoxic-ischemic brain injury, the chronic upregulation of cytokines and gliosis are also strongly associated with adverse neurodevelopmental outcomes [68,69]. Furthermore, intrauterine growth restriction is commonly caused by chronic placental insufficiency, and these infants often exhibit higher levels of proinflammatory cytokines in the blood [70,71]. However, it remains unclear whether neuroinflammation plays a direct role in the adverse neurological outcomes associated with intrauterine growth restriction or whether neuroinflammation is a secondary response to cellular injury. Nonspecific inflammation, involving elevated plasma concentrations of proinflammatory cytokines and neuroinflammation, can also occur following mechanical ventilation in preterm infants [72,73,74], with the duration of ventilation positively associated with the magnitude of the cytokine response. In turn, this increases the risk of developing brain injury, cerebral palsy, and neurodevelopmental delay [75,76]. Other potential nonspecific causes of perinatal neuroinflammation include intraventricular hemorrhage and cerebrospinal fluid circulation disorders such as post-hemorrhagic hydrocephalus, which are major complications of prematurity and closely associated with white matter injury and neurological disability [77,78,79]. For example, preterm infants with post-hemorrhagic ventricular dilatation were reported to show a marked and prolonged elevation in proinflammatory cytokines in the cerebrospinal fluid [80] and elevated microglial activation and cytokine release in the white matter [81].

## 5. Preterm Brain Injury

As described above, perinatal infection and inflammation are important risk factors for preterm brain injury. Cystic white matter injury is the most severe pattern of preterm brain injury and is characterized by multifocal areas of severe necrosis involving all cellular elements, including axonal degeneration, neuronal loss, and cyst formation, as well as widespread gliosis and myelination deficits [82,83,84]. Functionally, this pattern of injury gives rise to severe motor impairments (e.g., cerebral palsy) [85,86]. However, with modern advances in the detection of injury and neonatal care, the incidence and severity of cystic white matter injury has significantly decreased, accounting for <5% of cases in modern cohorts [69,87]. This has been replaced by a less severe but more diffuse pattern of white matter injury, which is observed in up to 79% of all preterm survivors [88]. Diffuse white matter injury is characterized as the acute death of pre-oligodendrocyte cells (preOLs) and more chronic deficits in oligodendrocyte maturation and axonal myelination, with concurrent reactive gliosis [89,90,91]. Diffuse white matter injury manifests as milder forms of neurodevelopmental impairment, including the mild impairment of motor skills and intellectual disabilities related to learning, cognition, attention, and socialization, which can persist into later life [92,93,94]. 

Importantly, preterm born infants also exhibit marked reductions in the growth and complexity of cortical and subcortical grey matter structures at term equivalence compared with healthy term counterparts [7,95], which persist into adulthood [96,97]. These deficits in grey matter development are associated with impaired cognition [98,99] and executive function [100,101,102]. In preterm born infants with severe cystic white matter injury, post-mortem studies suggest that these grey matter deficits involve the widespread death of neurons [103,104,105,106]. By contrast, in modern cohorts of preterm infants with diffuse white matter injury, there is limited evidence of neuronal loss [107]. Rather, more diffuse disturbances in neuronal dendritic growth and connectivity are suggested to underlie the deficits in grey matter development and cognitive function [108,109,110]. 

## 6. Molecular Mechanisms of Inflammation-Related Brain Injury

During development, neuroinflammation can have both beneficial and destructive consequences, influencing brain development. For example, under normal homeostatic conditions, cytokines and inflammatory cells such as astrocytes and microglia are involved in almost every major aspect of brain development and function, including synaptogenesis and refinement, apoptosis, and angiogenesis, as well as progenitor cell maintenance, proliferation, differentiation, and migration [111,112]. However, the activation of immune responses, either as a result of infection or sterile inflammation, can cause an imbalance in the production of cytokines and other immune molecules, which may propagate brain injury via mechanisms that can both directly and indirectly affect the survival and maturation of oligodendrocytes and neurons [113,114].

### 6.1. An Imbalance of Cytokines 

Cytokines are small molecular weight proteins involved in regulating inflammation and immune cell proliferation/differentiation, as well as providing crosstalk between the neural, endocrine, and immune systems [111]. Cytokines can be proinflammatory (e.g., TNF-α and IL-1), where they are involved in the upregulation of inflammatory responses, or anti-inflammatory (e.g., IL-4 and IL-10), where they act to regulate the proinflammatory cytokine responses [115]. In human preterm-born infants with evidence of brain injury, elevated concentrations of proinflammatory cytokines (e.g., TNF-α, IL-1β, IL-2, and IL-17) were reported in the amniotic fluid [116], umbilical cord blood [117], neonatal blood [118], cerebrospinal fluid [119], and brain tissue [120]. This elevation of proinflammatory cytokines is strongly correlated with adverse neurological outcomes [62,63,121]. Therefore, the negative effects of inflammation on brain development are considered to involve an imbalance between the pro- and anti-inflammatory cytokine responses [122,123].

Propagation of the systemic inflammatory response into the brain results in the local production of cytokines by central immune cells, brain endothelial cells, astrocytes, microglia, and neurons [124,125]. This central inflammatory response, secondary to systemic inflammation, can induce both direct and indirect injury to various CNS cells. For example, in vitro studies have shown that cytokine exposure can directly cause apoptosis [126,127], impaired maturation [128,129], and increased vulnerability to excitotoxic injury [130] of pre-oligodendrocytes and neural stem cells. Centrally produced cytokines can also activate microglia and astrocytes, which can induce brain injury via the various mechanisms described below. Cytokines have also been reported to induce brain injury by causing cerebral hypotension [131] and capillary thrombosis [132]. Furthermore, cytokines can indirectly alter brain development via the downstream modification of growth factors, including insulin-like growth factor 1 (IGF-1) [133,134] and brain derived neurotrophic factor [135,136]. Finally, centrally produced cytokines can further increase the BBB permeability, allowing the further entry of systemic cytokines, cytotoxic mediators, and other immune cells (e.g., leukocytes), providing a feedback loop for injury [137,138,139].

### 6.2. Microglia

Microglia are the primary immunocompetent cells of the CNS and play key roles in orchestrating central immune responses. Recent studies have demonstrated key roles for microglial in embryonic and postnatal brain development, including immunosurveillance, oligodendrocyte and neuronal development [140,141,142], and establishing brain circuits and connectivity [143,144]. During this period, microglia transition from an amoeboid shape (classically considered an “activated state”) to a more ramified morphology in an anatomical-, age-, and sex-dependent manner [145,146,147]. Following infection or inflammation, microglia in the brain can lose their normal homeostatic functions, becoming activated, which can further propagate the central inflammatory response and contribute to the evolution of preterm brain injury. For example, post-mortem studies of preterm-born infants with white matter injury show a marked upregulation of activated microglia [69,148]. In vitro, activated microglia can impair oligodendrocyte maturation via the excessive release of proinflammatory cytokines [149,150], free radicals (e.g., reactive oxygen species and nitric oxide synthase) [82,151], and excitotoxic molecules (e.g., glutamate) [152,153]. 

Experimental studies have reported that LPS-induced microglial activation heightens inflammatory responses, resulting in synapse loss, DNA fragmentation, and neuronal apoptosis [154,155]. Finally, activated microglia can indirectly induce injury to the developing brain by suppressing the production of various factors important for brain development, such as IGF-1 [156]. Of interest, in a mouse model of post-traumatic stress disorder, a recent study reported that activated microglia can also adopt a hyper-ramified state in response to neuroinflammation, which was associated with reduced spine density in the prefrontal cortex and hippocampus [157]. 

It is important to note that activated microglia show a wide range of phenotypes, which may play differing pathogenic versus protective/regenerative roles. Historically, microglia were broadly classified into two polarization states—the classically activated M1 polarization (considered cytotoxic and often associated with the propagation of inflammatory responses) or the alternatively activated M2a polarization (considered anti-inflammatory or tissue repair) and acquired deactivation M2b polarization (immune-regulatory state) [151,153,158,159,160]. However, this is now considered an oversimplification, as microglia can display a wide spectrum of activation states. Future studies are required to determine the specific roles, as well as the regional and temporal heterogeneity, of the various microglial phenotypes in clinically relevant animal models of perinatal brain injury. 

### 6.3. Astrocytes

Astrocytes are the most abundant cell type in the brain. Under normal physiological conditions, astrocytes play crucial roles in brain development and homeostasis, including the regulation of neuronal and oligodendrocyte development [161,162] and synaptogenesis [163]. As well as providing metabolic support for neurons [164], astrocytes play a role in the production of extracellular matrix components and trophic factors required for neuronal survival, oligodendrocyte survival and myelination [165,166], and BBB maintenance [167]. Importantly, astrocytes are considered to be immunocompetent cells, as they express receptors for both DAMPs and PAMPs and produce a variety of cytokines (e.g., TNF-α and interferon-γ) in response to infection or sterile inflammation [168,169]. 

In response to insults such as infection/inflammation or hypoxia-ischemia, astrocytes can become reactive (undergo significant hypertrophy and proliferation) and play an important role in the pathogenesis of preterm brain injury [170,171]. For example, post-mortem brains from preterm-born infants exhibit reactive astrocytes in areas overlapping with pre-oligodendrocyte maturation arrest [69,148]. In vitro, reactive astrocytes can impair oligodendrocyte maturation via the increased production of hyaluronan [172], bone morphogenetic protein [173,174], and platelet-derived growth factor [175]. In vivo studies have also shown that reactive astrocytes can impair oligodendrocyte maturation and remyelination by increasing Notch signaling [176], stimulating the Wnt/β-catenin signaling pathways [177], or stimulating cyclooxygenase-2-prostaglandin E2 signaling [178]. Additionally, reactive astrocytes can impair the development of neuronal dendrites and spines [179] and impair neuronal differentiation via the upregulation of interferon-induced transmembrane protein 3 [180]. Furthermore, reactive astrocytes produce a variety of proinflammatory cytokines that may amplify immune responses in the brain [181,182], inducing injury to oligodendrocytes and neurons. This amplification of the immune response was proposed to be related to impaired astrocytic communication. For example, TNF-α and IL-1β were reported to uncouple the astrocytic gap junction network but paradoxically enhance connexin hemichannel activity [183,184], which triggers intracellular Ca^2+^, Na^+^, and Cl^-^ influx, K^+^ efflux, and microglial activation [184,185]. Together, this increases the cellular permeability, resulting in an increased extracellular Ca^2+^ influx [186] and increased release of excitotoxic and inflammatory molecules/mediators [187], as well as the propagation of chronic inflammation via the inflammasome pathway (for review, see [188,189,190]). 

## 7. Anti-Inflammatory Agents in the Treatment of Inflammation-Related Brain Injury

**Figure 1:**
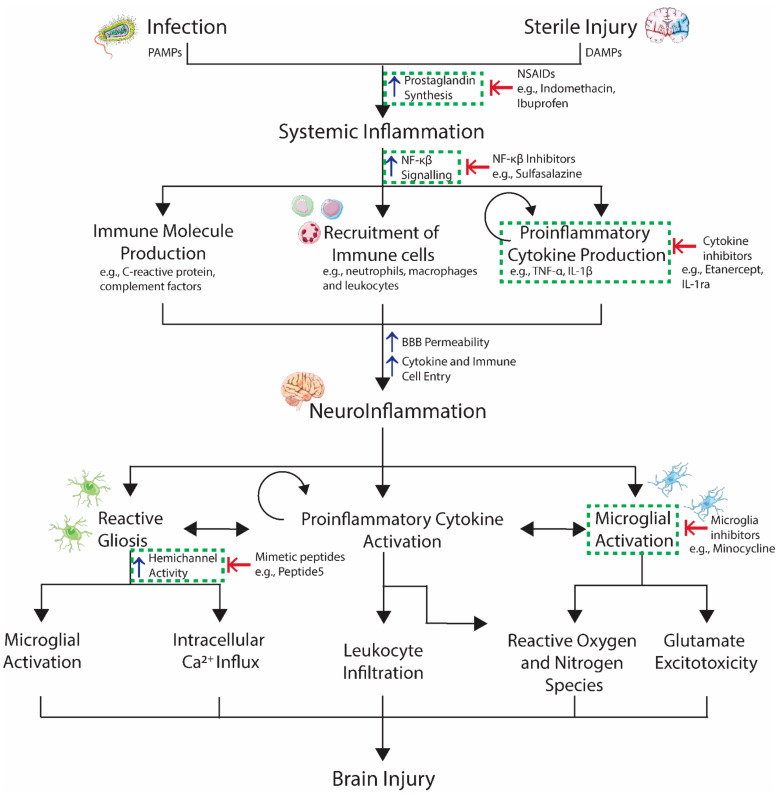
Propagation of systemic inflammation into the brain during development and the proposed pharmacological targets (dashed boxes) aimed at reducing immune responses peripherally and centrally. Abbreviations: PAMPs, pathogen-associated molecular patterns; DAMPs, damage-associated molecular patterns; NSAIDs, non-steroidal anti-inflammatory drugs; interleukin 1 beta; IL-1ra, interleukin-1 receptor antagonist; BBB, blood−brain barrier; and IGF-1, insulin-like growth factor 1. ↑, increase; ↓, decrease; ⇤, inhibitor.

Given the evidence for a causative role of systemic infection and nonspecific inflammation in preterm brain injury, with a likely mechanism involving the propagation of a central immune response, there is increasing interest in the use of treatments targeted towards reducing the immune response. Pharmacological interventions that range from the broad-spectrum inhibition of the inflammatory response to those that target specific neurotoxic immune molecules have been proposed as promising candidates for inflammatory-related brain injury (Figure 1). 

### 7.1. Inhibition of the Immune Response

Nonsteroidal anti-inflammatory drugs (NSAIDs) are a heterogeneous group of compounds that share analgesic and anti-inflammatory effects via the inhibition of cyclooxygenase (COX) activity, resulting in the inhibition of prostaglandin synthesis by immune cells [191,192]. COX-1 and COX-2 are key enzymes in the conversion of arachidonic acid to prostaglandins. COX-1 is constitutively expressed in most tissues, synthesizing prostaglandins that serve to maintain “housekeeping” functions (e.g., blood platelet production and regulating blood flow in the kidney and stomach) [193]. By contrast, COX-2 is undetectable under resting conditions and is primarily involved in the regulation of inflammatory responses (e.g., cytokine production) by synthesizing prostaglandin in response to various immune stimuli [194]. This COX-2-dependent production of prostaglandins has the potential to exacerbate brain injury, as in vitro studies have demonstrated that the induction of COX-2 activity can cause oligodendrocyte cell death [195] and neuronal injury [196]. At present, the NSAIDs indomethacin and ibuprofen are considered safe and well-tolerated by neonates and are currently administered during pregnancy for the prevention of preterm labor and for the treatment of persistent ductus arteriosus [197,198]. 

#### 7.1.1. Indomethacin

Indomethacin is a nonselective COX inhibitor, inhibiting both COX-1 (homeostatic function) and COX-2 (inflammatory function). Indomethacin is one of the most widely used NSAIDs in preterm-born infants (for the treatment of persistent ductus arteriosus). However, it remains unclear whether indomethacin can improve the mortality or neurodevelopmental outcomes in preterm-born infants. For example, when assessing the effect on mortality and neurodevelopmental outcomes in extremely low birth weight infants, the prophylactic administration of indomethacin was reported to reduce the frequency of severe periventricular and intraventricular hemorrhage [199]. In prospective cohort studies, the prolonged prophylactic administration of indomethacin was also associated with a reduced risk of white matter injury in infants born before 28 weeks of gestation [200,201]. Furthermore, the prolonged prophylactic administration of indomethacin to extremely preterm-born infants for the prevention of patent ductus arteriosus was reported to reduce white matter injury on subsequent MRI [200]. By contrast, a review of the Cochrane Neonatal database concluded that the prophylactic administration of indomethacin had no effect on the mortality or neurodevelopmental outcomes in preterm born infants [202]. In the Trial of Indomethacin Prophylaxis in Preterm Infants, the prophylactic administration of indomethacin did not reduce the burden of white matter injury, and the surviving infants exhibited moderate-to-severe cognitive delays at a corrected aged of 18 months [202]. Additionally, contraindications to indomethacin treatment include nephrotoxicity [203], adverse cerebral hemodynamics [204], and an increased risk of serious neonatal complications (e.g., intracranial hemorrhage, necrotizing enterocolitis, and patent ductus arteriosus) [202,205] in preterm infants born before 30 weeks of gestation.

**Figure 2:**
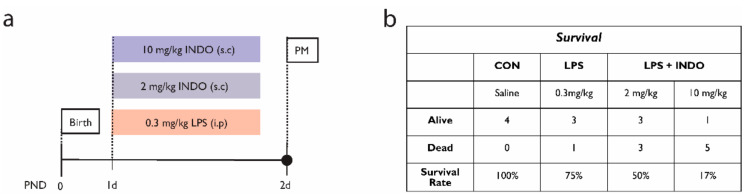
Administration of lipopolysaccharide (LPS) and indomethacin (INDO) in a neonatal rat model of inflammation-related brain injury. (**a**) Experimental design. Neonatal rats received single intraperitoneal (i.p.) injections of LPS or saline with concomitant subcutaneous (s.c.) injections of INDO or saline on postnatal day (PND)1 and were recovered to PND2. (**b**) A survival analysis and allocation of animals used in the dose-response study. Note that, because of the reduced survival associated with indomethacin, we were unable to assess the neurological outcomes. Gehan−Breslow−Wilcoxon test, *p* = 0.0317.

Experimental studies examining the neuroprotective effects of indomethacin for the treatment of perinatal brain injury are very limited. The prolonged prophylactic administration of indomethacin in postnatal day (PND)1–PND5 mice pups prior to the intracerebral administration of ibotenate abrogated the effects of IL-1β on excitotoxic lesions [206]. Additionally, treatment with indomethacin after hypoxic-ischemic brain injury in PND7 rat pups was associated with decreased caspase activity and the inhibition of glutathione depletion but aggravated lipid peroxidation [207]. To our knowledge, the use of indomethacin targeted at inflammation-related preterm brain injury has not been reported. Using a newborn rodent model of prolonged postnatal systemic inflammation [110], our preliminary data showed that a concomitant indomethacin treatment was associated with a marked increase in mortality (Figure 2). Speculatively, this may relate to an increased risk of sepsis [208] or adverse cardiovascular events (due to increased platelet aggregation and vascular tone) associated with indomethacin [209,210]. Thus, the administration of indomethacin during postnatal systemic inflammation may not be a viable treatment option. 

#### 7.1.2. Ibuprofen

Ibuprofen is another class of NSAID considered safe and well-tolerated by preterm infants. As for indomethacin, ibuprofen is a nonselective COX inhibitor, commonly used to treat patent ductus arteriosus. However, while the prophylactic use of ibuprofen is thought to have a better side effect profile than indomethacin, the clinical data are conflicting. For example, high-dose ibuprofen was associated with pulmonary hypotension [211], neonatal hyperbilirubinemia [212], and renal failure following oral administration [213]. By contrast, in combination with a Cochrane review, several studies have reported that prophylactic ibuprofen had no effect on the frequency of intraventricular hemorrhage [214,215,216] or neonatal mortality and morbidity [217] in very preterm-born infants. 

Despite these clinical findings, a number of experimental studies have demonstrated the neuroprotective effects of ibuprofen in various models of perinatal brain injury. For example, in a preterm-equivalent rat model of hypoxic-ischemic brain injury, the repeated administration of ibuprofen following injury attenuated the central inflammatory response, reduced pre-oligodendrocyte cell death [218], and attenuated injury to the serotonergic system [219]. In a neonatal piglet model of intrauterine growth restriction, repeated ibuprofen administration from PND1–PND3 was also associated with reduced inflammation in the parietal cortex and white matter, reduced cellular apoptosis, and the recovery of myelin staining and neuronal cell counts [220]. However, it is important to note, in these studies, that longer-term outcomes and neurobehavioral outcomes were not assessed. Furthermore, the neuroprotective effects of ibuprofen following infection and/or preclinical models of nonsterile inflammation remain unknown.

### 7.2. Inhibition of Cytokine Production

NF-κβ plays a key role in the immune response to infection by controlling the transcription of cytokines, chemokines, and adhesion molecules [221]. Thus, the pharmacological inhibition of NF-kβ transcriptional activity represents an important target for reducing inflammation. Sulfasalazine is a potent, specific inhibitor of NF-κβ transcription activity and has well-established anti-inflammatory and immunosuppressive actions [222]. Clinically, sulfasalazine is commonly used to treat inflammatory bowel disease [223] and is approved for use in pregnancy [224]. Sulfasalazine and its metabolite, sulfapyridine, readily cross the placenta, resulting in similar fetal and maternal concentrations [225]. However, very few studies have reported the neonatal outcomes following maternal exposure to sulfasalazine. In a Hungarian case-control study, there was no increase in the prevalence of congenital abnormalities in the children of women treated with sulfasalazine during pregnancy [224]. Similarly, in a meta-analysis, the exposure to sulfasalazine during pregnancy did not increase the risk of congenital malformations, stillbirths, spontaneous abortion, preterm labor, or low birth weight in the offspring [226]. 

Experimentally, only a limited number of studies have used sulfasalazine in animal models of inflammation-induced preterm birth and fetal hypoxia. However, it is important to note that these studies did not report any long-term outcomes or neuroprotective effects in the offspring. Of interest, in an ex vivo model of preterm birth that utilized human extraplacental membranes, sulfasalazine treatment blocked the LPS-induced production of cytokines and prostaglandins but increased the extent of apoptotic cell death in the chorionic membrane [227]. Furthermore, the administration of sulfasalazine in pregnant mice exposed to *Escherichia coli* was associated with reduced rates of preterm birth [228]. By contrast, in a model of fetal hypoxia, the prophylactic administration of sulfasalazine had no effect on mitigating the inflammatory response or improving the oxygenation [229]. However, given the crucial role of NF-kB signaling in neuronal and oligodendrocyte development [230,231], further studies are required to assess the safety profile and neurohistopathological outcomes associated with inhibiting NF-kβ transcriptional activity during early life exposure to inflammation.

### 7.3. Inhibition of Specific Cytokines

Numerous reports based on the extremely low gestational age newborns (ELGAN) study have demonstrated the association of elevated proinflammatory cytokines (e.g., IL-1β and TNF-α) in the cord blood or cerebrospinal fluid with brain injury and adverse neurodevelopmental outcomes in preterm-born infants [62,63,118,232]. Furthermore, in vitro studies have shown that proinflammatory cytokines such as TNF-α and IL-1β can induce oligodendrocyte [126] and neuronal [233] cell death, as well as impair oligodendrocyte [128] and neuronal [234] development. The systemic administration of specific cytokines such as IL-1β or TNF-α during the perinatal period was also associated with white and grey matter injury in various neonatal animal models [235,236,237]. Therefore, the inhibition of specific cytokines such as TNF-α or IL-1β may represent a further target for the mitigation of the cytokine response and reducing perinatal brain injury.

#### 7.3.1. TNF-α

Under normal conditions, TNF-α is expressed at low concentrations and acts as a potent regulator of developmental apoptosis and synaptogenesis [238]. Following immune activation, TNF-α concentrations are significantly upregulated in the systemic circulation [239] and in the brain [120]. Landmark human studies have also shown that elevated TNF-α concentrations following infection were associated with cognitive impairment in preterm infants [62,240], which persist into later life [241]. Therefore, TNF-α inhibition may be a viable treatment for inflammation-related preterm brain injury. 

Etanercept is a fusion protein consisting of the ligand-binding portion of the human p75 TNF receptor that effectively neutralizes the action of soluble TNF-α, thereby inhibiting TNF-α-induced proinflammatory signaling. While the use of etanercept to treat preterm brain injury has not been investigated clinically, etanercept is used to treat various inflammatory diseases, including rheumatoid arthritis, psoriatic arthritis, juvenile idiopathic arthritis, ankylosing spondylitis, and inflammatory bowel diseases. Although there have been no controlled trials during pregnancy, case series support the safety of etanercept for use during pregnancy [242,243,244], including no evidence of teratogenic risk when used in the first trimester [245,246]. In the Organization of Teratology Information Specialists rheumatoid arthritis pregnancy study, etanercept had no effect on the frequency of preterm birth or the incidence of major birth defects (8% for etanercept-exposed infants versus 5.7% for healthy control infants) [247]. Nevertheless, a number of cases of spontaneous abortion have been reported with etanercept use during pregnancy [248,249], although etanercept was used in combination with methotrexate, a chemotherapy agent and immunosuppressant. It is important to note that the clinical recommendations for etanercept use during pregnancy are made on a case-by-case basis [250]. Furthermore, the effects of the prolonged maternal etanercept use on neurodevelopmental outcomes of the offspring remain unclear. 

Experimental studies using TNF-α inhibitors to treat prematurity and perinatal brain injury show conflicting findings. For example, in pregnant mice, inhibiting TNF-α activity was reported to have no effect in preventing preterm labor or prolonging pregnancy [251]. By contrast, inhibiting TNF-α using antibodies against TNF-α or etanercept reduced fetal deaths and the rate of preterm delivery in an endotoxin-induced mouse model of preterm delivery [252] and significantly lowered blood pressure in response to placental ischemia in rodents [253]. Etanercept administration was also associated with reduced neuronal apoptosis and synaptic loss, and improved long-term cognitive outcomes, following propofol-induced neurotoxicity in neonatal rats [254], largely abolished cerebral TNF-α production and brain injury following inflammatory and excitotoxic brain injuries in mice [123], and improved white matter myelination in septic neonatal rats [255]. In addition, concurrent etanercept administration reduced white matter astrogliosis after acute on chronic LPS exposure in preterm fetal sheep [256]. However, in a neonatal rat model of inflammation-related preterm brain injury, the coadministration of LPS and TNF-α antibody at PND5 had no effect on LPS-induced brain injury [257]. Furthermore, the prophylactic administration of etanercept did not protect the brain from excitotoxic brain injury in neonatal mice [123]. Together, these contrasting findings associated with inhibiting the activity of TNF-α likely reflect the complex actions of TNF-α during brain development, in addition to the species variability between studies and variability in the induction of brain injury, optimal dosing, and the timing of treatment. 

#### 7.3.2. IL-1

IL-1α and IL-1β are key proinflammatory cytokines that mediate both central and systemic inflammatory responses, as well as playing key roles in modulating synaptic plasticity [258] and lipid metabolism [259]. Under normal conditions, IL-1 expression is very low in the brain. However, in response to systemic and central inflammation or injury, there is a marked upregulation of IL-1 (particularly, IL-1β), which has been proposed to play a role in the pathogenesis of preterm brain injury. For example, the systemic administration of IL-1β was associated with preterm labor and delivery in pregnant mice [260], decreased pup survival [261], and fetal brain cortical thinning in the offspring [262]. A repeated postnatal injection of IL-1β was associated with oligodendrocyte dysmaturation, persisting myelination deficits, and impaired cognition in neonatal mice [235]. 

The IL-1 receptor antagonist (IL-1Ra) is an endogenous ligand that binds to the IL-1R, limiting the proinflammatory actions of IL-1α and IL-1β. Clinically, IL-1Ra is used for the treatment of rheumatoid arthritis [263] and neonatal-onset multisystem inflammatory disease [264], although no effect of IL-1Ra was found in the treatment of severe sepsis in adults [265]. Clinically, IL-1 has also been implicated in preterm labor [266]. However, there are conflicting results from experimental studies. In pregnant mice, the treatment with human recombinant IL-1Ra prevented IL-1-induced preterm birth [267], but had no effect on endotoxin-induced preterm delivery [251]. Interestingly, the effects of IL-1Ra administration in the treatment of perinatal brain injury are more consistent. For example, in mice, repeated antenatal treatment with the noncompetitive IL-1R inhibitor, 101.10, following the maternal administration of LPS during mid-gestation was reported to prevent neonatal mortality and fetal brain inflammation, without altering the postnatal growth [268]. In a maternal model of inflammation induced by LPS exposure, the coadministration of LPS and recombinant human IL-Ra to pregnant rats reduced the fetal mortality, cell death, and behavioral outcomes [269]. Similarly, blocking IL-1 receptor activity attenuated the inflammation-related white matter injury following postnatal exposure to LPS in neonatal rats [257] and preserved the motor functions and exploratory behaviors after intrauterine LPS exposure and postnatal hypoxia-ischemia [270]. 

### 7.4. Inhibition of Microglial Activation

As described above, activation of the microglia forms part of the neuroinflammatory cascade following fetal and early postnatal infection/inflammation and is thought to play a key role in potentiating preterm brain injury. As such, there is strong interest in the development and use of pharmacological inhibitors of microglial activation to limit their proinflammatory and cytotoxic mechanisms. Minocycline is an FDA-approved semisynthetic broad-spectrum tetracycline antibiotic used clinically to treat bacterial infections [271], acne [272], and rheumatoid arthritis [273]. In addition, despite a small sample size, minocycline for the treatment of multiple sclerosis was reported to be well-tolerated and decreased the annual relapse rate [274]. Importantly, despite not being a selective microglia inhibitor, minocycline can cross the BBB and exerts potent anti-inflammatory effects by decreasing microglial activation [275]. 

Experimentally, minocycline treatment is beneficial in various animal models of perinatal brain injury. For example, the administration of minocycline immediately before or after cerebral hypoxia-ischemia in neonatal rodents can reduce neuronal apoptosis [276] and pre-oligodendrocyte cell death [277], preserve the integrity of the central serotonergic network [278], and improve the neurobehavioral outcomes [279]. These neuroprotective actions were related to the inhibition of microglial-induced activation of the mitogen-activated protein kinase pathway [280] and microglial-induced oxidative and nitrosative stress [281]. Minocycline has also shown promise in the treatment of inflammation-related brain injury. For example, in neonatal rats, the administration of minocycline before and after LPS exposure from PND5–PND8 was associated with reduced ventricular enlargement, decreased cell death, the recovery of myelin basic protein staining [282], and improved behavioral outcomes [283]. Delayed minocycline treatment following intrauterine exposure to the Gram-positive mimetic polyriboinosinic-polyribocytidylic acid in early gestation [284] or postnatal LPS exposure [285] in mice also inhibited microglial activation in the offspring and was associated with improved cognitive outcomes. However, there are some contrasting findings. For example, minocycline treatment following cerebral hypoxia-ischemia was neuroprotective in neonatal rats but increased injury in neonatal mice [286]. The perinatal [287] and postnatal [288] administration of minocycline in normal mice caused widespread cell death in the primary sensory cortex, septum, hippocampus, and hypothalamus. Similarly, minocycline was associated with reduced cytokine production and the suppression of oligodendrocyte maturation and neurogenesis during the normal development in neonatal rats [289], highlighting the important role of inflammation during normal brain development. Overall, these contrasting findings may be related to the broad-spectrum inhibition of the different microglial phenotypes (e.g., inhibiting both toxic and neuroprotective phenotypes). Therefore, therapeutic interventions that specifically block toxic microglial phenotypes may have the greatest potential to protect the injured brain. 

### 7.5. Inhibition of Astrocyte Activation

As described in Section 6.3, in response to non-physiological immune stimulation (e.g., following cell death) or inflammation, astrocytes can start rapidly proliferating and exhibit a reactive phenotype [111,170,171], while reactive astrocytes can impair pre-oligodendrocyte and neuronal maturation [178,180]. Importantly, human post-mortem studies have shown that reactive astrogliosis is a hallmark feature of white matter injury in preterm-born infants [69]. Thus, targeting astrocyte reactivity may be a promising therapeutic strategy. 

One potential mechanism by which astrocyte activation can contribute to brain injury is through abnormal signaling via hemichannels and gap junctions. Both infectious and sterile inflammation can trigger the abnormal opening and activity of astrocytic hemichannels (e.g., connexin 43 [Cx43]), paradoxically reducing astrocyte gap junction communication [290]. These changes may compromise the ability of astrocytes to maintain homeostasis, resulting in the abnormal regulation of cellular Ca^2+^, glucose uptake, and release of excitotoxic molecules. Peptide5 is a connexin mimetic peptide that targets the extracellular loop of the Cx43 molecule, preventing hemichannel opening without affecting the gap junction communication [291], which, in turn, has been proposed to reduce hemichannel-mediated adenosine triphosphate release and activation of the inflammasome pathway [292]. A treatment with peptide5 following recovery from global cerebral ischemia in near-term fetal sheep was associated with a marked reduction in seizure activity and improved neuronal and oligodendrocyte survival [293,294,295,296]. Similarly, peptide5 infusion following umbilical cord occlusion in preterm fetal sheep was associated with improved oligodendrocyte maturation [297]. However, neuroprotection was not observed when peptide5 infusion was administered during global cerebral ischemia [294] or at higher doses [298]. The neuroprotective actions of blocking Cx43 activity have yet to be assessed in a neonatal model of inflammation-related brain injury.

## 8. Concluding Remarks

Currently, no neuroprotective strategies have been proven to prevent inflammation-related brain injury in preterm-born infants. Although there are a handful of clinical situations in which pregnant mothers or newborn infants are administered an anti-inflammatory agent, their effects on neurodevelopmental outcomes seem to be limited. It is important to consider that, in the clinic, the timing of infection or inflammation is often unclear. These events are induced by a wide range of infectious agents and nonspecific inflammatory events that can occur both prenatally and postnatally, with both acute and chronic time courses. Critically, any clinically relevant therapy must be effective when administered well after the onset of inflammation and also consider the potential for disrupting the physiological role of inflammation in normal brain development. Thus, the development of clinically effective neuroprotective anti-inflammatory agents requires concerted preclinical studies that systematically address these issues and, in particular, include a detailed understanding of the therapeutic window of opportunity.

## Data Availability

Not applicable.
